# Simulation-based training of endoscopic hemostasis for Japanese pediatric endoscopy learners: a pilot program

**DOI:** 10.1016/j.igie.2024.04.003

**Published:** 2024-04-09

**Authors:** Takeshi Kanno, Itaru Iwama, Yutaka Hatayama, Suguo Suzuki, Yutaro Arata, Tomoyuki Koike, Atsushi Masamune

**Affiliations:** 1 Division of Gastroenterology, Tohoku University Graduate School of Medicine, Sendai, Japan; 2 R & D Division of Career Education for Medical Professionals, Medical Education Center, Jichi Medical University, Shimotsuke, Japan; 3 Division of Gastroenterology and Hepatology, Saitama Prefectural Children's Medical Center, Saitama, Japan; 4 Graduate Medical Education Center, Tohoku University Hospital, Sendai, Japan

## Abstract

**Background and Aims:**

Endoscopic hemostasis training, often in emergencies, can be challenging for pediatric endoscopists. This study aimed to establish hands-on seminar sessions using the novel clip hemostasis simulator and to explore the underlying concerns about endoscopic hemostasis among pediatric endoscopists and the potential of simulator-based training (SBT).

**Methods:**

An SBT course was incorporated into seminars by the Japanese Society for Pediatric Gastroenterology, Hepatology, and Nutrition. Surveys using the visual analog scale (VAS; 0-100) completed by consenting pediatricians during 4 seminars from October 2021 to March 2023 were compared with responses of adult GI residents and junior residents.

**Results:**

Fifty-two pediatric endoscopists (median age, 31 year; postgraduate year 7) were enrolled. A median VAS score of 47 (interquartile range [IQR], 23.5-65) for understanding endoscopic hemostasis was significantly lower than that of adult GI residents (median, 76; IQR, 58-82; *P* < .001) and comparable with junior residents (median, 54; IQR, 50-65). Pediatric endoscopists' confidence in independently performing hemostasis was low, with a median score of 0 (IQR, 0-16.5), which was below adult GI residents (median, 67; IQR, 49-77; *P* < .001) and junior residents (median, 11.5; IQR, 10-39; *P* = .014). Regarding skill enhancement by SBT, a median score of 94.5 showed high and no significant difference from junior residents and adult GI residents, respectively. All pediatric endoscopists expressed an interest in repeated SBT sessions.

**Conclusions:**

Pediatric endoscopists were concerned about their competence in endoscopic hemostasis. The simplified SBT programs with the simulator may potentially improve their skills and confidence. (Clinical trial registration number: UMIN000035735.)

GI bleeding represents a critical emergency, not only in adults but also in pediatric patients. Upper GI bleeding (UGIB) accounts for 10% to 15% of all pediatric hospitalizations,^1^ with 6% to 25% of these cases requiring admission to the intensive care unit.^2^ The etiology of UGIB in children varies with age, but gastroduodenal ulcers are the most common cause, accounting for 30% to 50% of pediatric UGIB cases.^1^^,^^3^^,^^4^

Endoscopic hemostasis for peptic ulcer was recommended following adult treatment protocols with thermal or mechanical methods.^5^^,^^6^ Hemostatic clips are widely used in many cases as a representative mechanical method.^1^^,^^4^ A report of 17 pediatric patients with nonvariceal UGIB who underwent endoscopic hemostasis showed a recurrent bleeding rate of 40%.^7^ Although this is a small sample size and could not be directly compared, it contrasts with the 5% to 8% rate of recurrent bleeding after endoscopic hemostasis reported in adults,^8^^,^^9^ suggesting a greater risk of recurrent bleeding in pediatric cases that requires careful attention.

Globally, particularly in North America, training programs for pediatric endoscopists are being developed, although their effectiveness remains unclear.^10^ Furthermore, training in invasive procedures like endoscopic hemostasis for pediatric endoscopists is not implemented sufficiently worldwide. In Japan, systematic programs have not been established, and the current approach often involves collaboration with adult endoscopists on a case-by-case basis.

We developed a dry simulator, named the Medical Rising STAR-Ulcer type (Denka Company Limited, Tokyo, Japan), designed to replicate spurting bleeding similar to Forrest Ia from multiple exposed blood vessels at the base of an ulcer using a specially formulated elastic resin, allowing for clip hemostasis or coagulation with a hemostatic grasper.^11^ The ulcer model can be attached anywhere on the stomach or duodenal bulb. A dry simulator that allows the use of actual endoscopic and therapeutic devices could provide more practical training than virtual reality simulators. This research focused on creating hands-on seminar sessions using the novel hemostasis simulator to investigate pediatric endoscopists' concerns and expectations regarding endoscopic hemostasis and its simulation-based training (SBT).

## Methods

From October 2021, an SBT program that incorporated an endoscopic hemostasis training course using a simulator as part of hands-on seminars was conducted by the Japanese Society for Pediatric Gastroenterology, Hepatology, and Nutrition. This hands-on seminar included screening EGD and targeted biopsy sampling training sessions with the other dry simulator models and insertion method sessions of colonoscopy with another dry simulator. Pediatric endoscopy learners who joined the SBT session and orally consented to participate in our survey were recruited. After finishing the hands-on SBT session, participants completed a questionnaire with a visual analog scale (VAS) and multiple-choice questions. The questionnaire included background information (sex, age, and years after graduation) and questions on understanding the endoscopic hemostasis procedure, confidence in achievement of the treatment without a supervisor, the simulator's replicability of a bleeding ulcer compared with an actual patient, and reliability of the SBT session with the novel model (Table 1).

**Table 1:** List of questions

Questions	Measurement
Understanding	
1. Do you understand the procedure of endoscopic hemostasis?	VAS (0-100)
Confidence	
2. Are you confident that you can stop bleeding on your own without help from the supervising staff physician?	VAS (0-100)
Evaluation of simulation-based training of endoscopic hemostasis with the novel model	
3. Was this simulator an accurate depiction of bleeding in a real patient?	VAS (0-100)
4. Do you believe this simulator can enhance your endoscopic hemostasis skills?	VAS (0-100)
5. Would you recommend this simulator to your colleagues?	VAS (0-100)
6. Would you like to train again using this simulator? (no more, every 3-6 months, or about once a month)	Multiple choice

*VAS*, Visual analog scale.

For a comparative analysis, we added a junior resident group to represent novice learners of endoscopy and an adult GI resident group. Junior residents had undergone a similar SBT program as that of the pediatric endoscopy trainees. In this study, “junior residents” refers to doctors in their first or second year after graduation undergoing foundational medical training, whereas “GI residents” are those who have completed their junior residency and are specializing in gastroenterology, typically in their third to sixth postgraduate year, working toward acquiring their specialty certification.

In a previous international collaborative study, we evaluated the learning effects of simulators on 50 GI residents from Canada and Japan using questionnaires administered before and after SBT sessions.^12^ For the comparison group in the current study, we included 39 Japanese GI residents. This group consisted of 38 residents from the previous study who completed post-SBT questionnaires, plus 1 additional Japanese GI resident who underwent SBT after the completion of the previous study recruitment, for a total of 39 Japanese GI residents.

In response to considerations of potential bias because of the different timing of hands-on seminars for pediatric endoscopists, junior residents, and GI residents, it is pertinent to mention that all training sessions were conducted by the same trainers (either T.Kanno or Y.H.). This approach ensured that the core instructional content remained consistent across all groups. Specifically, participants across all groups practiced basic endoscopic hemostasis using a bleeding ulcer model in the antral area of the greater curvature of the stomach. Advanced training on the upper body posterior wall of the stomach was offered to all GI residents as part of their SBT, whereas for pediatric endoscopic learners and junior residents, such advanced training was offered selectively based on the individual's ability to perform stable endoscopic procedures and rapidly achieve hemostasis in the antral area. For the training, GIF H260 (Olympus Corp, Tokyo, Japan) and GIF H290 (Olympus Corp) endoscopes and HX-610-135 (Olympus Corp) hemostatic clips were used.

Statistical comparisons were performed using the Mann-Whitney U test with adjustments for multiple comparisons made using the Bonferroni method. A *P* < .017 defined a significant difference because of Bonferroni correction. Analyses were performed using StatsDirect statistical software, version 3.3.5 (StatsDirect Ltd, Wirral, UK). The study protocol was approved by the Ethics Committee of Tohoku University (no. 2018-1-780). The study was conducted in accordance with the Declaration of Helsinki and good practice guidelines and was registered with the UMIN Clinical Trials Registry (registration no. UMIN000035735).

## Results

### Simplified training for endoscopic hemostasis with hemostatic clips

In our hands-on sessions, we focused on pediatricians with limited endoscopic experience, aiming to demystify endoscopic hemostasis using hemostatic clips and ensuring that learners grasped the essential elements of the procedure. Using the Rising-STAR-Ulcer type, which allows for the placement of ulcer lesions at any location, we positioned the model in the greater curvature of the antral area of the stomach. In EGD, navigating the scope along the greater curvature is typically positioned in this area at the 6 o'clock view, allowing minimal left–right angle manipulation and easier creation of a stable view primarily through up–down angle manipulation.

**Figure 1:**
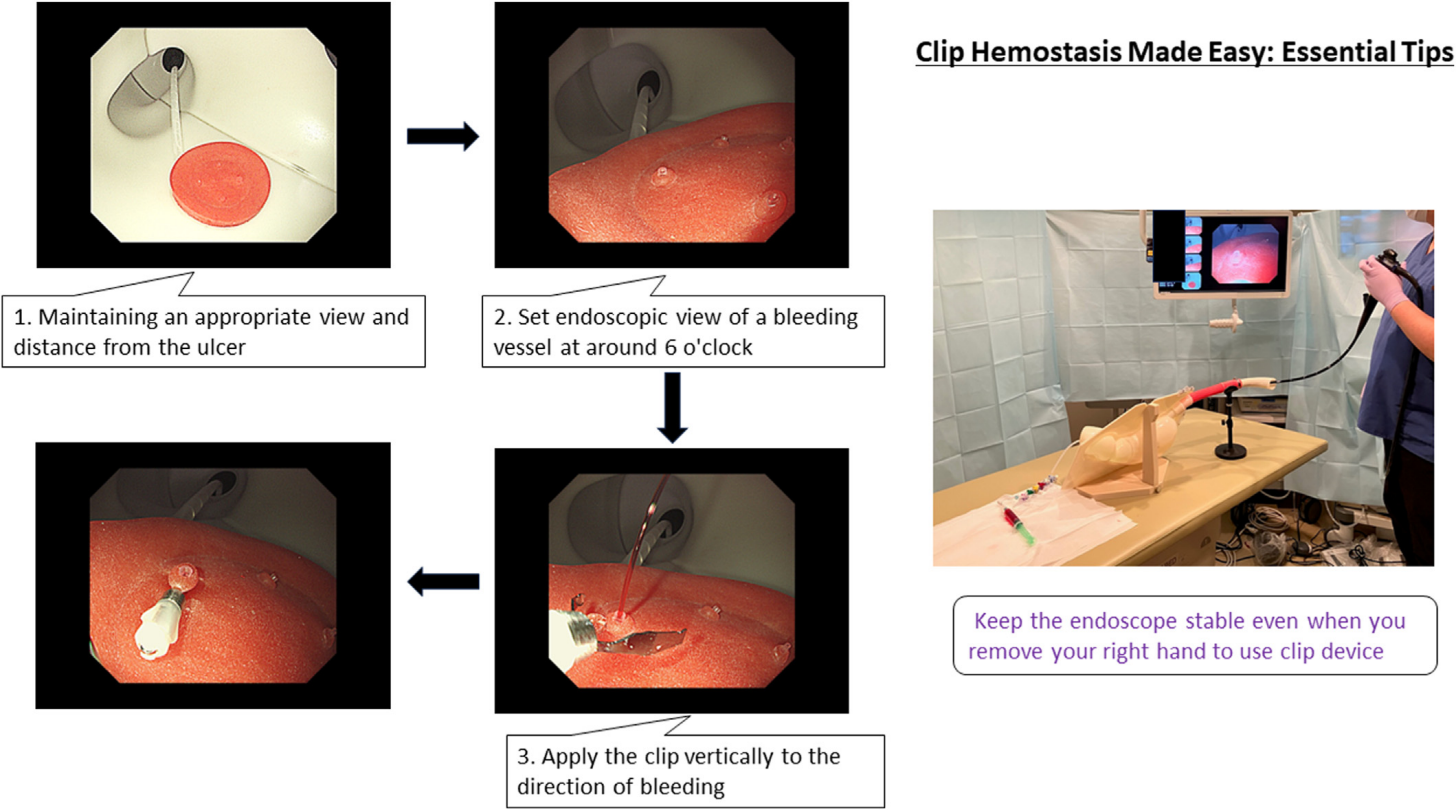
Overview of the simulator model and essential tips for endoscopic hemostasis with hemostatic clips.

During the training, we emphasized 3 key tips (Fig. 1). The first focused on maintaining an appropriate view and distance from the ulcer for clear visualization and accurate targeting. The second tip involved setting the endoscopic view of a bleeding vessel at around 6 o'clock, simplifying intervention with up–down angulation. We also highlighted the importance of left-hand stabilization of the endoscope while operating the device with the right hand, which is crucial for making a stable approach to the bleeding point. The third tip was to apply the clip vertically to the direction of bleeding, facilitating easier grasping and simplifying the clipping process compared with a precise pinpoint application. Participants practiced on 2 exposed vessels with real-time feedback. This training course not only helped to deconstruct the hemostasis technique into manageable elements, but also aimed to foster success in less-experienced trainees.

### Participant characteristics and questionnaire results

Table 2 presents the background characteristics of 52 pediatric endoscopists who participated in the seminar. Their median age was 31 years (35 men and 17 women, median postgraduate year 7). For comparative analysis, data from control groups were included: 10 junior residents (median age, 26 years; 7 men and 3 women; median postgraduate year 1) and 39 adult GI residents (median age, 31 years; 32 men and 7 women; median postgraduate year 6).

**Table 2:** Baseline characteristics of participants

Characteristics	Pediatric endoscopists (n = 52)	Junior resident (n = 10)	Adult GI residents (n = 39)
Median age, y (range)	31 (26-49)	26 (24-32)	31 (27-35)
Sex, man/woman	35/17	7/3	32/7
Median postgraduate year (range)	7 (2-24)	1 (1-2)	6 (3-9)

**Figure 2:**
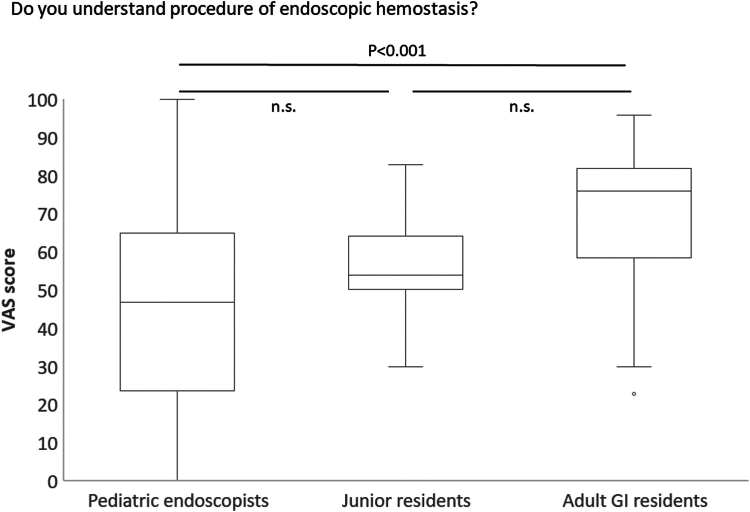
Comparison by VAS score among the 3 groups for understanding the technique of endoscopic hemostasis. The median is shown as the mid-point of the data and the *line* dividing the box 2 parts for each group. The *box* represents the middle 50% of values for each group. Seventy-five percent of the scores fall below the upper quartile, and 25% of scores fall below the lower quartile. *VAS*, visual analog scale.

For understanding the technique, for the question, “Do you understand the procedure of endoscopic hemostasis?,” pediatric endoscopists recorded a median VAS score of 47 (interquartile range [IQR], 23.5-65), which was significantly lower than the median score of 76 (IQR, 58-82; *P* < .001) for adult GI residents (Fig. 2). The median score for junior residents was 54 (IQR, 50-65), showing no significant difference from pediatric endoscopists. The scores between junior residents and adult GI residents did not reach a significant difference by the Mann-Whitney U test with Bonferroni correction (*P* = .023 [>.017]) (Fig. 2).

**Figure 3:**
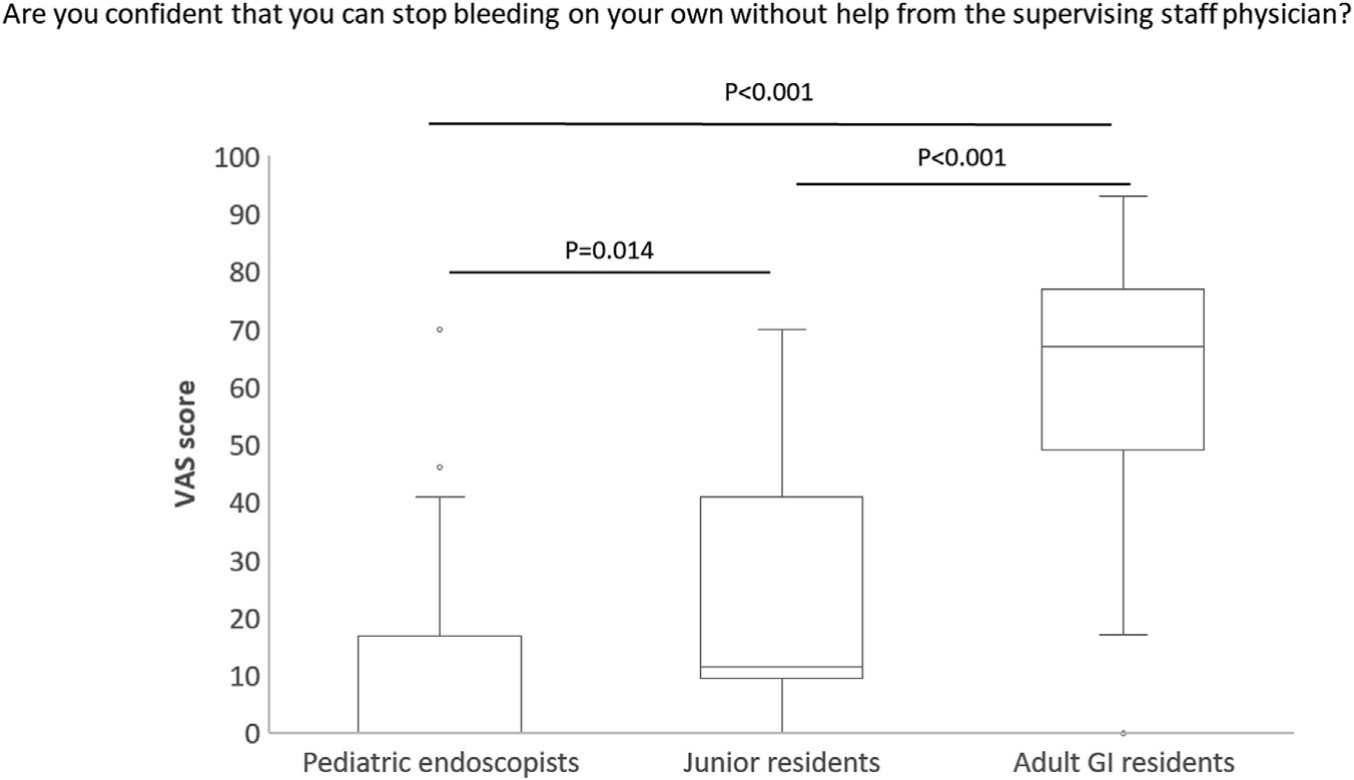
Comparison by VAS score among the 3 groups for confidence in performing endoscopic hemostasis independently. The median is shown as the mid-point of the data and the *line* dividing the *box* into 2 parts for each group. The *box* represents the middle 50% of values for each group. Seventy-five percent of the scores fall below the upper quartile, and 25% of the scores fall below the lower quartile. *VAS*, visual analog scale.

Regarding confidence in performing the procedure, for the question, “Are you confident that you can stop bleeding on your own without help from the supervising staff physician?,” the median VAS score for pediatric endoscopists was 0 (IQR, 0-16.5). This score was not only significantly lower than the median of 67 (IQR, 49-77) for adult GI residents (*P* < .001), but was also significantly lower than the median of 11.5 (IQR, 10-39) for junior residents (*P* = .014). Furthermore, the VAS score for junior residents was significantly lower compared with adult GI residents (*P* < .001) (Fig. 3).

Table 3 details the SBT evaluations using VAS scores. For the simulator's realism (“Was this simulator an accurate depiction of bleeding in a real patient?”), median VAS scores were 79.5 for pediatric endoscopists, 66 for junior residents, and 86 for adult GI residents, with no significant group differences. Regarding skill enhancement (“Do you believe this simulator can enhance your endoscopic hemostasis skills?”), scores were 94.5, 82, and 89, respectively, again showing no significant variance. For recommending the simulator (“Would you recommend this simulator to your colleagues?”), the scores were uniformly high (97, 92.5, and 87 for pediatric endoscopists, junior residents, and adult GI residents, respectively) across all groups, indicating a broad consensus without significant disparities. In response to the multiple-choice question, “Would you like to train again using this simulator?,” all 52 pediatric endoscopists expressed a desire for future SBT sessions, with 69.2% (36/52) indicating a preference for monthly training.

**Table 3:** Evaluation of simulation-based training of endoscopic hemostasis with the novel model

Questions	Pediatric endoscopists (n = 52)	Junior residents (n = 10)	Adult GI residents (n = 39)
3 Was this simulator an accurate depiction of bleeding in a real patient?	79.5 (67-96.5)	66 (45-82)	86 (72-95)
4 Do you believe this simulator can enhance your endoscopic hemostasis skills?	94.5 (78-100)	82 (75-99)	89 (80-98)
5 Would you recommend this simulator to your colleagues?	97 (79.5-100)	92.5 (77-100)	87 (80-98)

Value are median (interquartile range). For all 3 questions, the Mann-Whitney U test with the Bonferroni correction showed no significant differences.

## Discussion

This study explored the learning needs and appropriate training methods for inexperienced pediatric endoscopy learners. We compared the survey results of pediatricians who participated in a pediatric endoscopy hands-on seminar with those from adult GI residents, who have relatively more endoscopy experience, and junior residents, representing endoscopy novices. The findings revealed that pediatric endoscopists rated their understanding of endoscopic hemostasis lower than adult GI residents and expressed significantly less confidence in performing hemostasis without a supervisor, even compared with junior residents. A French study found an incidence of UGIB in children of about 1 to 2 cases per 10,000 annually.^13^ Furthermore, the etiology included peptic ulcers, esophageal variceal rupture, and Mallory-Weiss syndrome—conditions seen in both adults and children—as well as issues more unique to pediatrics, like foreign body ingestion and congenital hemophilia.^1^ This rarity, coupled with a variety of causes, means that sufficient cases could be possible only in high-volume centers, which may be influenced by the lower confidence levels observed among pediatric endoscopists. In fact, several participants expressed concerns in the free-text comments regarding the scarcity of learning opportunities. This feedback underscores the necessity for further investigation into the background level of learning and experience among pediatric endoscopists, junior residents, and GI residents in a qualitative manner.

Although there is a global trend toward establishing systematic training programs for pediatric endoscopists, Japan is still in the process of developing such effective course programs. In this context, seminars such as those organized by the Japanese Society for Pediatric Gastroenterology, Hepatology, and Nutrition provide valuable opportunities for learning and reflection. Of course, it is important to recognize that not all cases should be solved by pediatric endoscopists alone. Understanding the basic elements of endoscopic hemostasis as an initial response and recognizing the existence of more complex cases creates the opportunity for appropriate collaboration with adult gastroenterologists. The clip-based endoscopic hemostasis technique, as demonstrated in Figure 1, simplifies fundamental skills such as identifying the bleeding point, keeping an appropriate view, and operating the clipping device in a stable manner. After acquiring such fundamental skills, it may be useful to set up a more advanced SBT targeting duodenal ulcers, which are considered more difficult to treat. Evaluations of the SBT showed consistently high efficacy ratings across all groups, suggesting that it may be a particularly beneficial educational tool for pediatric endoscopists as primary training who are often limited by less hands-on experience. This aligns with previous research that SBT had the potential to contribute to the current competency-based model of training.^14^

In our previous study with gastroenterology residents experienced in endoscopic therapy, we found a significant decrease in the success rate of hemostasis for lesions on the upper posterior wall of the stomach compared with those on the greater curvature of the antral area.^12^ This finding highlights the ability of the simulator to replicate more complex scenarios. Considering that all participating pediatric endoscopists expressed interest in repeating the SBT, this suggests the potential for developing advanced course programs. Such SBT programs could be designed not only for novices, but also for more advanced learners as a subsequent step.

Future studies would benefit from directly comparing the Medical Rising STAR model model with established training models, such as the CompactEASIE model, known for its use of porcine organs in simulating GI bleeding.^15^ Such comparative evaluations are crucial for determining the relative effectiveness and identifying the unique advantages of each model, thereby enhancing endoscopic training methodologies.

This study has several limitations that should be acknowledged. First, our assessment was based only on evaluations conducted at a single point after the training, which does not capture the long-term learning effects. Future program development should include both subjective and objective evaluations before and after the program, as well as over time, to effectively measure learning outcomes. Second, the small sample size of junior residents (n = 10) may not fully represent the perspectives of all novices, although their inclusion alongside adult GI residents, who are similar in age to pediatric endoscopists but more experienced, provides valuable insights for comparative analysis. Furthermore, our study did not collect information in a format that allows for a comparative analysis of the training levels or experiences among pediatric endoscopists, junior residents, and GI residents attending the hands-on seminars. Finally, the lumen part of the simulator model used was designed based on an adult stomach under air inflation, which may have a simplified field of view acquisition compared with pediatric conditions. However, this lower level of difficulty was considered acceptable for the purpose of learning simplified endoscopic hemostasis skills for novices.

In conclusion, learners of pediatric endoscopy had concerns about their understanding and technical confidence in independently performing endoscopic hemostasis. The simplified learning program with the novel simulator showed the potential to contribute to improvements in these areas.

## Patient consent

We did not include patients into this study.

## Disclosure

The following author disclosed financial relationships: T. Kanno: Research support from Denka Company Limited (JAPAN). All other authors disclosed no financial relationships. T. Kanno and Y. Arata received research support for this study was provided by 10.13039/501100001691JSPS KAKENHI (grant no. 22K10460).
